# *Chaenomelis fructus* inhibits osteoclast differentiation by suppressing NFATc1 expression and prevents ovariectomy-induced osteoporosis

**DOI:** 10.1186/s12906-020-2841-9

**Published:** 2020-02-05

**Authors:** Minsun Kim, Ho-Seok Kim, Jae-Hyun Kim, Eun-Young Kim, Bina Lee, Sung Yub Lee, Jae-Yun Jun, Min Beom Kim, Youngjoo Sohn, Hyuk-Sang Jung

**Affiliations:** Department of Anatomy, College of Korean Medicine, Kyung Hee University, Seoul, 02447 Republic of Korea

**Keywords:** Chaenomelis Fructus, Osteoclast, RANKL, NFATc1, Postmenopausal osteoporosis

## Abstract

**Background:**

Osteoporosis is related to the number and activity of osteoclasts. The goal of the present study was to demonstrate the effect of Chaenomelis Fructus (CF) on osteoclastogenesis and its mechanism of bone loss prevention in an OVX-induced osteoporosis model.

**Methods:**

Osteoclasts were induced by RANKL in RAW 264.7 cells. TRAP assay was performed to measure the inhibitory effect of CF on osteoclast differentiation. Then, Expression of nuclear factor of activated T-cells (NFATc1), c-Fos which are essential transcription factors in osteoclastogenesis were detected using western blot and RT-PCR. The osteoclast-related markers were measured by RT-PCR. Moreover, the ability of CF to inhibit bone loss was researched by ovariectomized (OVX)-induced osteoporosis.

**Results:**

Cell experiments showed that CF inhibited osteoclast differentiation and its function. Immunoblot analyses demonstrated that CF suppressed osteoclastogenesis through the NFATc1 and c-Fos signaling pathways. RT-PCR determined that CF inhibited osteoclast-related markers, such as tartrate-resistant acid phosphatase (TRAP), cathepsin K (CTK), osteoclast-associated immunoglobulin-like receptor (OSCAR), ATPase H+ Transporting V0 Subunit D2 (ATP6v0d2) and carbonic anhydrase II (CA2). In animal experiments, CF showed an inhibitory effect on bone density reduction through OVX. Hematoxylin and eosin (H&E) staining analysis data showed that CF inhibited OVX-induced trabecular area loss. TRAP staining and immunohistochemical staining analysis data showed that CF displayed an inhibitory effect on osteoclast differentiation through NFATc1 inhibition in femoral tissue.

**Conclusion:**

Based on the results of in vivo and in vitro experiments, CF inhibited the RANKL-induced osteoclasts differentiation and its function and effectively ameliorated OVX-induced osteoporosis rats.

## Background

Osteoporosis is an important health problem for the elderly. The prevalence is increasing worldwide due to the aging population, and diagnosis and treatment remain challenging [1]. Osteoporosis is metabolic bone disease characterized by reduction in bone mass, low bone strength and microstructure deterioration in trabecular and cortical skeleton, possibly leading to a higher risk of fracture [2]. Osteoporosis is divided into primary and secondary; postmenopausal osteoporosis is the most common of the primary forms. The quantity and quality of bone are maintained in a delicate balance between bone resorption by osteoclasts and bone formation by osteoblasts [3]. However, hormone deficiency causes abnormal activation of osteoclasts and breaks the balance of bone metabolism [4].

Osteoclasts are large multinucleated cells derived from hematopoietic precursor cells [5]. Progression of differentiation is regulated by the indicator of osteoblast-secreted factors: receptor activator of nuclear factor Kappa-B ligand (RANKL) [6]. RANKL binds to its receptor (RANK) on osteoclast precursor cell surfaces, and TNF receptor associated factor 6 (TRAF6) is activated [7]. Then, TRAF6 stimulates the two main transcription factors in osteoclast differentiation, nuclear factor of activated T cells c1 (NFATc1) and c-Fos [8, 9]. These factors control the expression of osteoclast function-related genes and proteins such as tartrate-resistant acid phosphatase (TRAP/acp5), cathepsin K (CTK), matrix metallopeptidase 9 (MMP-9), osteoclast-associated receptor (OSCAR), ATP6v0d2 and carbonic anhydrase II (CAII) [10, 11].

Currently, various drugs are used in the clinic to treat osteoporosis, such as bisphosphonates [12], denosumab [13], teriparatide [14] and estrogen-like drugs. However, long-term administration of these drugs is not unsuitable because they cause unwanted side effects such as breast cancer and endometrial cancer in patients [15]. Herbal medicine has relatively few side effects and is suitable for chronic diseases requiring long-term treatment. *Chaenomelis Fructus* (CF) is the dried fruit of *Chaenomeles sinensis* Koehne, which is a medicine traditionally used in East Asian countries such as Korea, China, and Japan. In oriental medicine, CF has been used as a remedy for patients with weak muscles and bones, muscle pain, and arthritis [16]. Moreover, recent studies have shown that CF components have an anti-inflammatory effect, which is an effective treatment for arthritis [17]. Many studies have reported that inflammation is associated with osteoclasts [18, 19]. Therefore, we expect that CF would be effective in the treatment of osteoclasts. However, the effects of CF on osteoclasts and osteoporosis have not been studied.

In the present study, we investigated the effects of CF on osteoclastogenesis in RAW 264.7 cells and demonstrated their mechanism of action. We also examined whether CF ameliorates ovariectomy (OVX)-induced osteoporosis in rats.

## Materials and methods

### Reagents

RANKL was purchased from Peprotech (London, UK). Alpha-minimum essential media (α-MEM), fetal bovine serum (FBS), penicillin/streptomycin (P/S) and Dulbecco’s phosphate buffered saline (DPBS) were obtained from Gibco (Gaithersburg, NY, USA). TRAP assay kit was obtained from Sigma Aldrich (Saint Louis, MO, USA). Osteo assay surface multiple well plates were obtained from Corning, Inc. (New York, NY, USA). Anti-c-Fos antibody, anti-TRAF6 antibody and anti-β-actin antibody were obtained from Santa Cruz (CA, USA). Anti-NFATc1 antibody was purchased from BD Pharmingen (San Diego, CA, USA). Anti-MMP-9 antibody and anti-CTK antibody were purchased from Abcam (Cambridge, MA, USA). Anti-total-ERK antibody, anti-phospho ERK antibody, Anti-total-JNK antibody, anti-phospho JNK antibody, Anti-total-p38 antibody and anti-phospho p38 antibody were purchased from Cell signaling (Beverly, MA, USA). Anti-NFATc1 antibody was purchased from BD Pharmingen (San Diego, CA, USA).PCR primers were obtained from Genotech (Daejeon, Korea). All of the chemicals used in the experiments were of analytical grade or complied with the level required for cell culture.

### Preparation of CF

CF was received from the Kyung Hee University Medical Center. Professor Yungmin Bu at the Herbology Laboratory, College of Korean Medicine, Kyung Hee University corroborated the CF. CF was extracted by heating in distilled water for 2 h, filtered using gauze and filter paper, and lyophilized. The extracted powder was stored at − 20 °C and diluted with water before use. The yield was 20.5%. A voucher specimen of the plant material used in this study has been deposited in the department of anatomy herbarium [KHU-ANA-A068].

### Analysis of CF extract with HPLC

Standard stock solutions (1 mg/ml) of Chlorogenic acid (Sigma-Aldrich, Saint-Louis, MI, USA) were prepared in methanol. A Waters 2695 system equipped with a Waters 2487 Dual λ absorbance detector was used for the analysis of both chlorogenic acid and chlorogenic acid from CF as the standard. The separation was carried out on an Xbridge-C18 (250 mm × 4.6 mm, 5 μm) with a C18 guard column. The binary mobile phase consisted of solvent A, Acetonitrile, and solvent B, water containing 1% acetic acid. All the solvents were filtered through a 0.45 μm filter prior to use. The volume injected is 10 μl. The elution conditions were 0–40 min. Chlorogenic acid was detected at 325 nm.

### RAW 264.7 cell culture and cell viability

RAW 264.7 cells were purchased from Korean Cell Line Bank (Seoul, Korea). Cells were cultured in DMEM with 10% FBS and 1% P/S. The cells were maintained in a humidified atmosphere containing 5% CO_2_ at 37 °C. To evaluate the toxicity of CF, RAW 264.7 cells at 5 × 10^3^ cells per well were seeded on a 96-well plate and then treated with various concentrations of CF (1, 10, 100 μg/ml) for 24 h. Cell viability was measured using the MTS assay kit (Promega, WI, USA) according to the manufacturer’s protocol.

### TRAP staining and pit formation assay

To investigate the effect of CF on osteoclastogenesis, RAW 264.7 cells at 5 × 10^3^ cells per well were seeded on a 96-well plate in α-MEM and then treated with RANKL (100 ng/ml) and various concentrations of CF (1, 10, 100 μg/ml). Five days later, cells were fixed using a 10% formalin solution and stained for TRAP according to the manufacturer’s protocol. The stained cells were imaged with an inverted microscope. Cells were considered to be osteoclasts if they were TRAP-positive multinucleated cells with more than 5 nuclei. TRAP activity was determined in the supernatants using a TRAP solution (4.93 mg Pnpp in 0.5 M 750 ml acetate solution, dissolved with 150 ml tartrate acid solution). After 1 h, the reaction was terminated with 0.5 M NaOH, and the absorbance was measured at a 405-nm wavelength using an enzyme-linked immunoassay reader (ELISA, Versamax, Molecular Devices, CA, USA). To investigate the effect of CF on bone resorption activity, RAW 264.7 cells at 5 × 10^3^ cells per well were seeded on an osteo assay surface multiple well plate and then treated with RANKL (100 ng/ml) and various concentrations of CF (1, 10, 100 μg/ml). Five days later, cells were removed with 4% NaClO. The pit area was imaged using an inverted microscope (100×) and was measured using ImageJ software (National Institutes of Health, Bethesda, MD, USA).

### Western blot analysis

RAW 264.7 cells at 2 × 10^5^ cells per well were seeded on a 60-mm plate and then treated with RANKL (100 ng/ml) and various concentrations of CF (1, 10, 100 μg/ml). Protein was prepared using RIPA buffer (50 mM Tris-Cl, 150 mM NaCl, 1% NP-40, 0.5% sodium deoxycholate, 0.1% SDS, protease inhibitor cocktail, and phosphatase inhibitor cocktail). Concentrations of protein were determined using the bicinchoninic acid method (Thermo Scientific, Pittsburgh, PA, USA). The protein extracts (10~30 μg) were loaded on SDS–polyacrylamide gel and transferred to a nitrocellulose membrane (Whatman, Dassel, Germany). The membrane was blocked with 5% skim milk for 1 h. Next, the membrane was incubated overnight at 4 °C in a 1:1000 dilution of each primary antibody in a 1% BSA solution and then probed for 1 h with secondary antibodies (Jackson ImmunoResearch, West Grove, PA, USA). Bands of interest were detected using an enhanced chemiluminescence detection system (Santa Cruz).

### Reverse transcription polymerase chain reaction (RT-PCR) analysis

The total RNA was isolated from TRIzol (TaKaRa Bio, Otsu, Japan), and cDNA was synthesized from the total RNA using the PrimeScript RT Reagent Kit (Takara Biotechnology, Otsu, Japan). Reverse transcription reactions were performed with the Invitrogen Reverse-transcription Reagent Kit (Invitrogen, Carlsbad, CA, USA), and PCR was performed with the MG Taq DNA polymerase (MG med, Seoul, Korea). The PCR conditions were as follows: 95 °C for 30 s, 55–58 °C for 30 s, and 72 °C for 30 s for 30–40 cycles. All experiments proceeded according to the manufacturer’s instructions. The primers used in this experiment were as follows: Mouse *NFATc1*: forward, TGC TCC TCC TCC TGC TGC TC and reverse, CGT CTT CCA CCT CCA CGT CG; Mouse *c-Fos*: forward, ATG GGC TCT CCT GTC AAC AC and reverse, GGC TGC CAA AAT AAA CTC CA; Mouse *RANK*: forward, AAA CCT TGG ACC AAC TGC AC and reverse, ACC ATC TTC TCC TCC CHA GT; Mouse *TRAP*: forward, ACT TCC CCA GCC CTT ACT ACC G and reverse, TCA GCA CAT AGC CCA CAC CG; Mouse *CTK*: forward, AGG CGG CTA TAT GAC CAC TG and reverse, CCG AGC CAA GAG AGC ATA TC; Mouse *OSCAR*: forward, CTG CTG GTA ACG GAT CAG CTC CCC AGA and reverse, CCA AGG AGC CAG AAC CTT CGA AAC T; Mouse *ATP6v0d2*: forward, ATG GGG CCT TGC AAA AGA AAT CTG and reverse, CGA CAG CGT CAA ACA AAG GCT TGT A; Mouse *CAII*: forward, CTC TCA GGA CAA TGC AGT GCT GA and reverse, ATC CAG GTC ACA CAT TCC AGC A; Mouse *GAPDH*: forward, ACT TTG TCA AGC TCA TTT CC and reverse, TGC AGC GAA CTT TAT TGA TG. Gene expression levels were normalized to glyceraldehyde-3-phosphate dehydrogenase (GAPDH).

### Animal and OVX-induced osteoporosis model and treatment

In vivo experiments were approved by the Institutional Animal Care and Use Committee at the Kyung Hee University Laboratory Animal Center (approval number: KHUASP (SE)-13–051). Forty 12-week-old, healthy, female Sprague-Dawley (SD) rats (Nara biotech, Seoul, Korea) were acclimatized for one week. After anesthesia with isoflurane, both ovaries were removed to complete a postmenopausal osteoporosis model. In addition, the sham-operated group was given the same surgical stress, but the ovaries were not ablated. The rats were randomly divided into 5 groups of 8 rats as follows:

(1) sham-operated + distilled water via oral administration;

(2) OVX + distilled water via oral administration;

(3) OVX + 17β-estradiol (E_2_, 100 μg/kg) via oral administration;

(4) OVX + low concentration of CF (CF-L, 35 mg/kg) via oral administration; and.

(5) OVX + high concentration of CF (CF-H, 350 mg/kg) via oral administration. Oral administration was carried out every morning for 8 weeks. At the end of the treatment, rats were anesthetized with pentobarbital sodium (80 mg/kg) and sacrificed by collecting blood close to the lethal dose with a cardiac puncture. The ovaries and femur were collected and weighed. Femurs were fixed with 10% neutral buffered formalin (NBF) overnight and were then stored at room temperature until used in the experiment.

### Measurement of serum biomarkers

Blood was collected through the left ventricle. Serum was prepared by centrifugation and then stored at − 80 °C. TRAP activity in serum was measured according to the described in the method section. Measurement of ALP, AST and ALT in serum was performed by DKKorea Inc. (Seoul, Korea) with ELISA instrument.

### Measurement of bone density

The collected femur was placed in a conical tube and filled with distilled water. To remove the air inside the femur, the conical tube was kept in a vacuum for 90 min. Water on the femur was completely removed with gauze and weighed. The femur was transferred to a new conical tube and filled with distilled water. The femur was reweighed in the water. As previously described, bone density was calculated using Archimedes’ principle (g/cm^3^ bone volume) [20].

### Histological staining

Femurs were fixed in 10% NBF and decalcified in 10% ethylene diamine tetraacetate (EDTA) for 3 weeks and embedded in paraffin. The paraffin-embedded femurs were cut into 5-μm-thick sections on a microtome (ZEISS, Oberkochen, GERMANY) with a disposable blade. Femur sections were stained with hematoxylin and eosin (H&E) for histopathological changes. To identify osteoclasts in the femoral tissues, TRAP staining was performed. TRAP staining was carried out according to the manufacturer’s manual and specimens were then counterstained with methyl green. The stained tissues were visualized with a light microscope (DP73, Olympus, Tokyo, Japan) (200×).

### Immunohistochemical (IHC) staining

The paraffin-embedded femurs were cut into 5-μm-thick sections on a microtome. After endogenous peroxidase was blocked using 3% H_2_O_2_/Mt-OH for 15 min at room temperature, for blocking, normal serum (Gibco, Gaithersburg, MD, USA) was reacted at room temperature for 30 min. The sections were incubated with the primary antibody anti-NFATc1 (1:100) at 4 °C overnight. The tissues were then incubated in secondary antibody (1:100 biotinylated) for 60 min at room temperature. Finally, sections were stained with 3,3′-diaminobenzidine (Vector Labs, Burlingame, CA, USA) and then counterstained with hematoxylin. The stained-tissues were visualized with a light microscope (200×).

### Statistical analysis

All data are expressed as the means ± SEM in at least three or more experiments. Statistical significance was evaluated for any differences among the groups by one-way ANOVA, followed by Dunnett’s multiple comparison test. *P* values < 0.05 were considered significant.

## Results

### Quality assessment of the CF extract

**Fig. 1:**
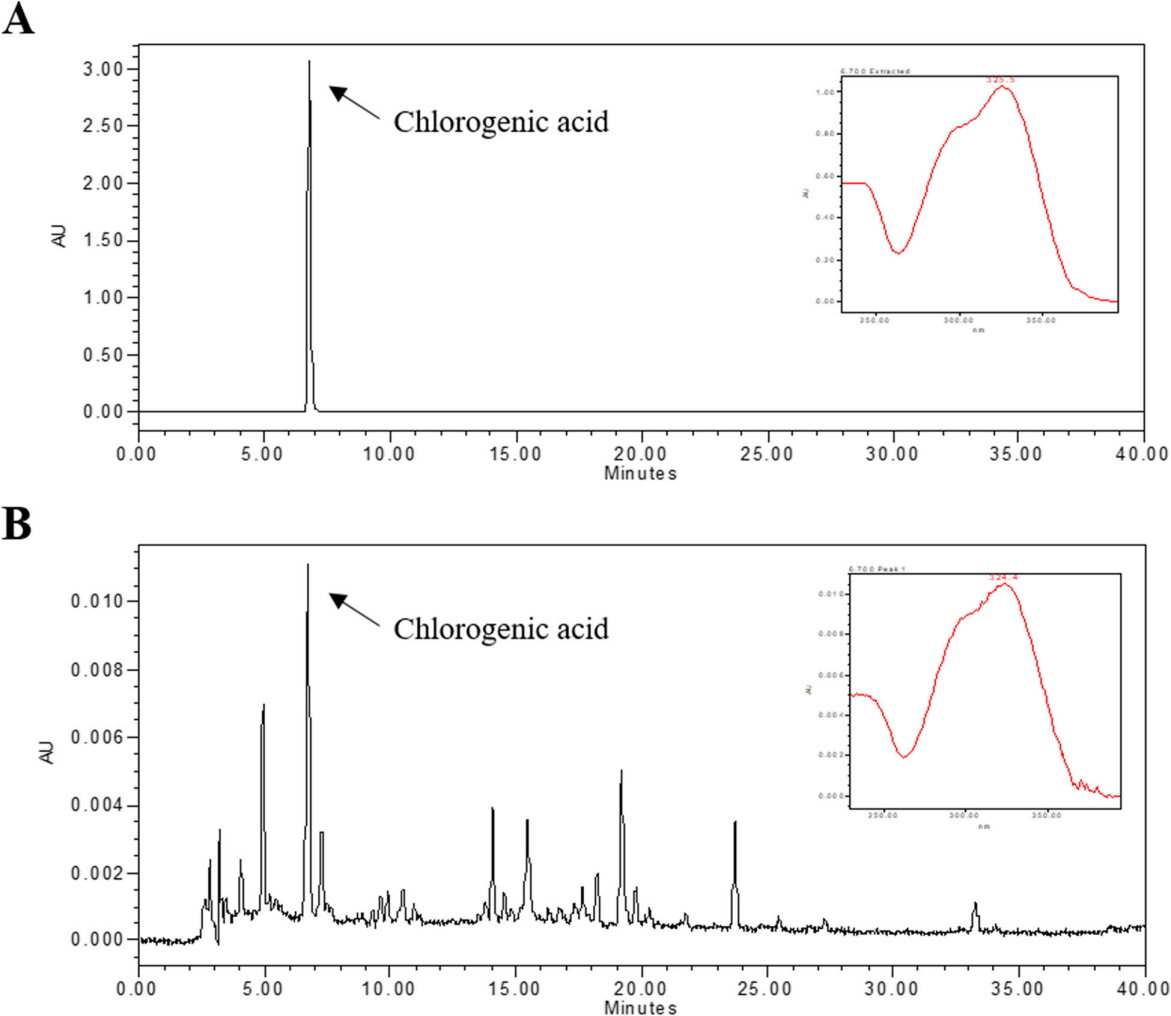
HPLC Chromatograms of the **a** Chlorogenic acid and **b** CF

Chlorogenic acid is a marker compound for the authentication of CF [21]. The chromatogram of the water extract from CF showed many peaks at a retention time of 0 and 40 min, and chlorogenic acid was found at the same retention time as the standards (Fig. 1).

### Effect of CF on osteoclastogenesis and resorptive activity

**Fig. 2:**
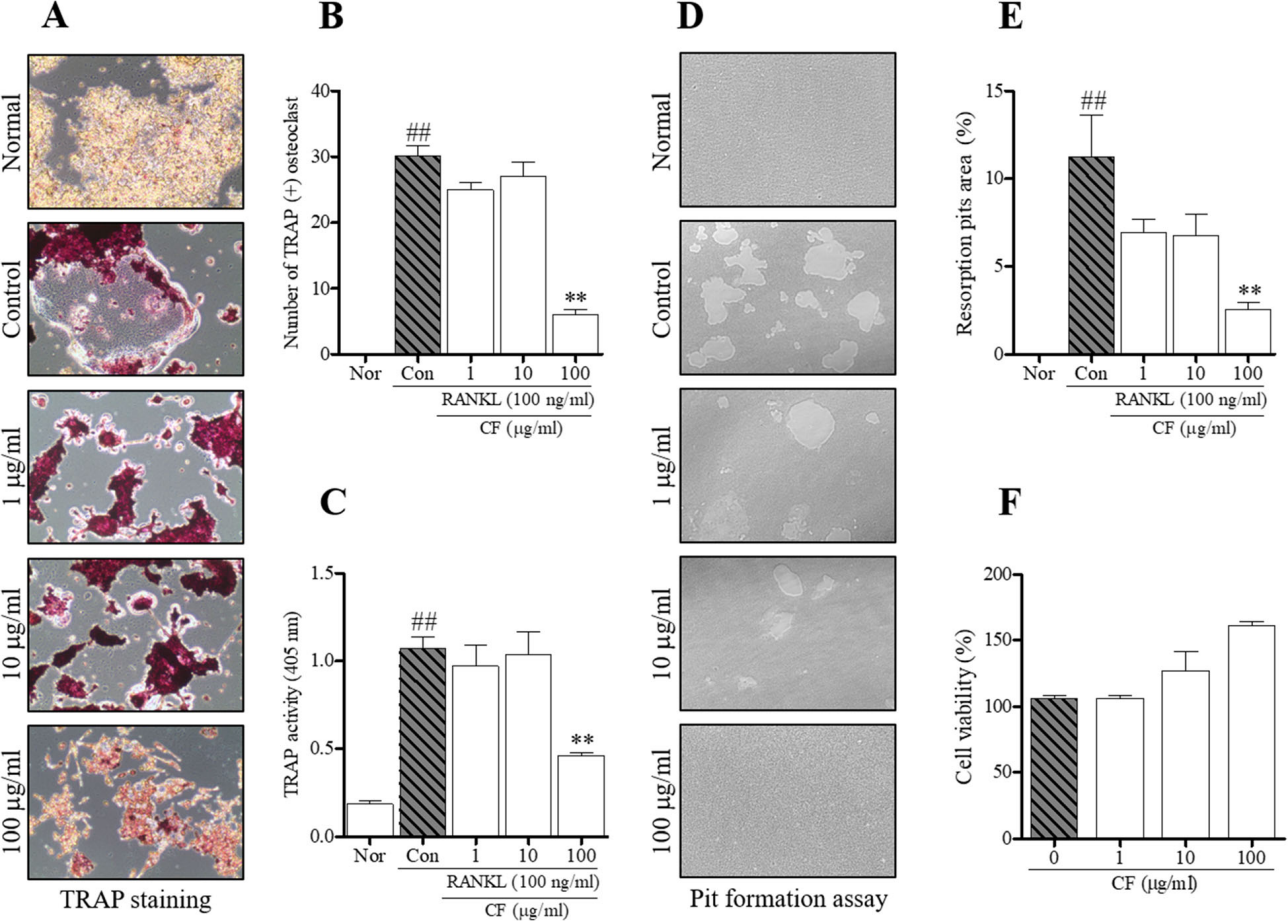
The effects of CF on RANKL-induced osteoclastogenesis and bone resorptive activity in RAW 264.7 cells. **a** TRAP-positive cells were captured using an inverted microscope (100×). **b** TRAP-positive cells were counted, and **c** TRAP activity was measured by an ELISA reader. **d** Pit area was captured using an inverted microscope (100×). **e** Pit area was measured using ImageJ software. **f** The cell viability of RAW 264.7 cells in the presence of various concentrations of CF was determined by MTS assay. Data represent the means ± SEM of three independent experiments. ^##^*P* < 0.01 compared with normal and ^**^P < 0.01 compared with control. TRAP, tartrate-resistant acid phosphatase; RANKL, receptor activator of nuclear factor kappa β ligand

To demonstrate the effect of osteoclast differentiation of CF, we used a general in vitro method of RANKL treatment in RAW 264.7 cells [22]. As shown in Fig. 2a-c, CF significantly inhibited osteoclast differentiation and activity at a concentration of 100 μg/ml (*P* < 0.01). Similarly, CF decreased the pit area (Fig. 2d), and its area was measured using ImageJ (*P* < 0.01) (Fig. 2e). Then, to confirm the cell cytotoxicity of CF, the effects of different concentrations (1, 10, 100 μg/ml) of CF on the survival rate of RAW 264.7 cells were confirmed by MTS assay. The concentration of CF used in this experiment did not affect the cell viability (Fig. 2f).

### CF inhibits expression of TRAF6, translocation of NF-κB and phosphorylation of JNK and p38

**Fig. 3:**
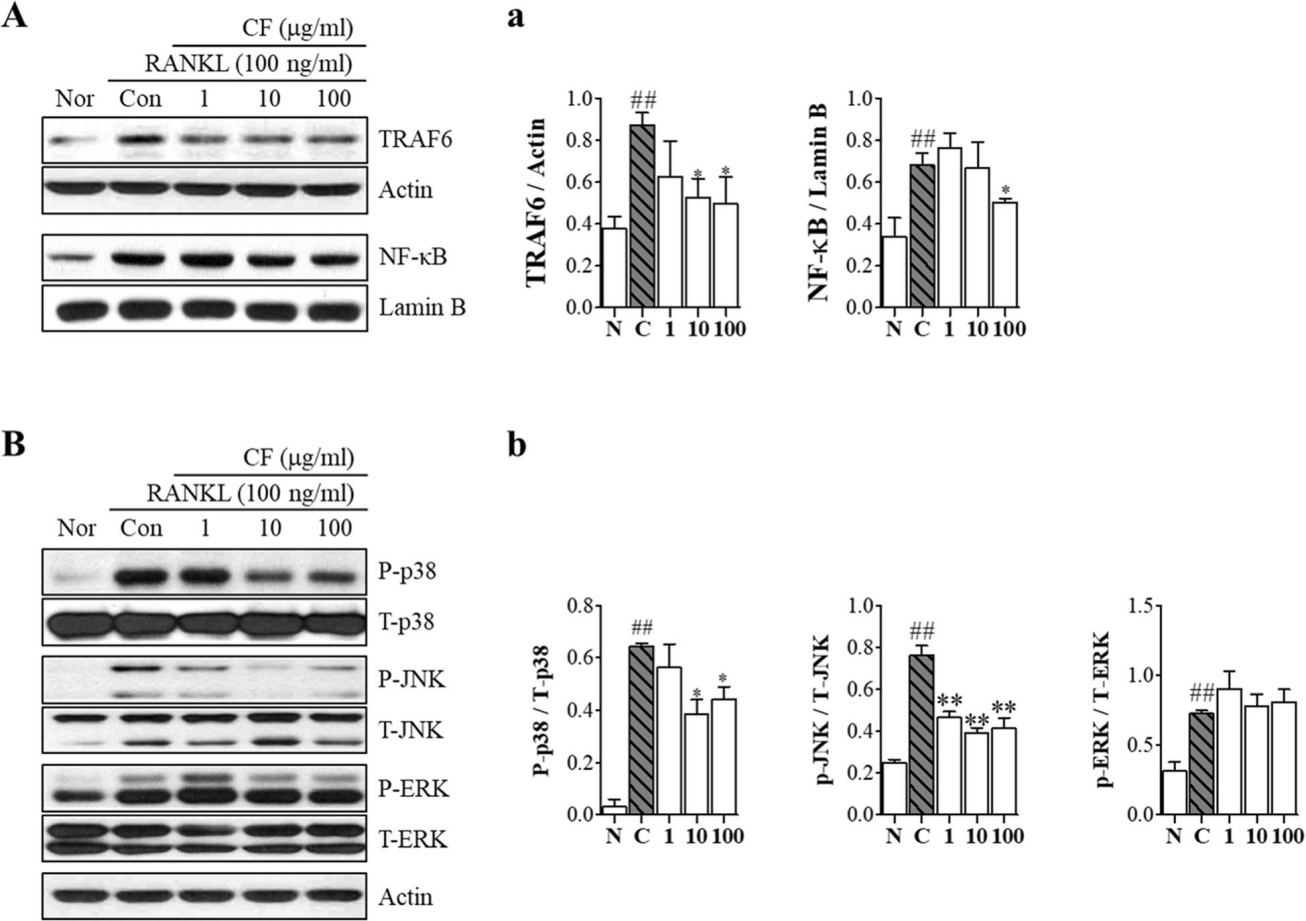
The effects of CF on TRAF6, NF- κB and MAPK expression. RAW 264.7 cells were treated with RANKL (100 ng/ml) and CF (1, 10, 100 μg/ml) for 30 min. **A** TRAF6 and NF- κB expression were detected using western blot. **a** Protein expression was normalized to actin. **B** Phosphorylation of MAPKs were determined by western blot. **b** Protein expression was normalized to each total form. Data represent the means ± SEM of three independent experiments. ^##^*P* < 0.01 compared with normal and ^**^P < 0.01, ^*^P < 0.05 compared with control. TRAF6, tumor necrosis factor receptor-associated factor 6; NF- κB, nuclear factor-kappa B; MAPKs, Mitogen-activated protein kinase. ERK, extracellular-signal-regulated kinase; JNK, c-Jun N-terminal kinases; Nor, nontreated; Con, RANKL-treated control

To further confirm the molecular mechanism of CF in osteoclast differentiation, we confirmed the expression of TRAF6, NF-κB and MAPKs. CF inhibited the expression of TRAF6 and translocation of NF-κB (*P* < 0.05) (Fig. 3A and a). Moreover, CF inhibited the phosphorylation of p38 (*P* < 0.05) and JNK (*P* < 0.01) (Fig. 3B and b).

### CF inhibits RANKL-induced expression of NFATc1 and c-Fos

**Fig. 4:**
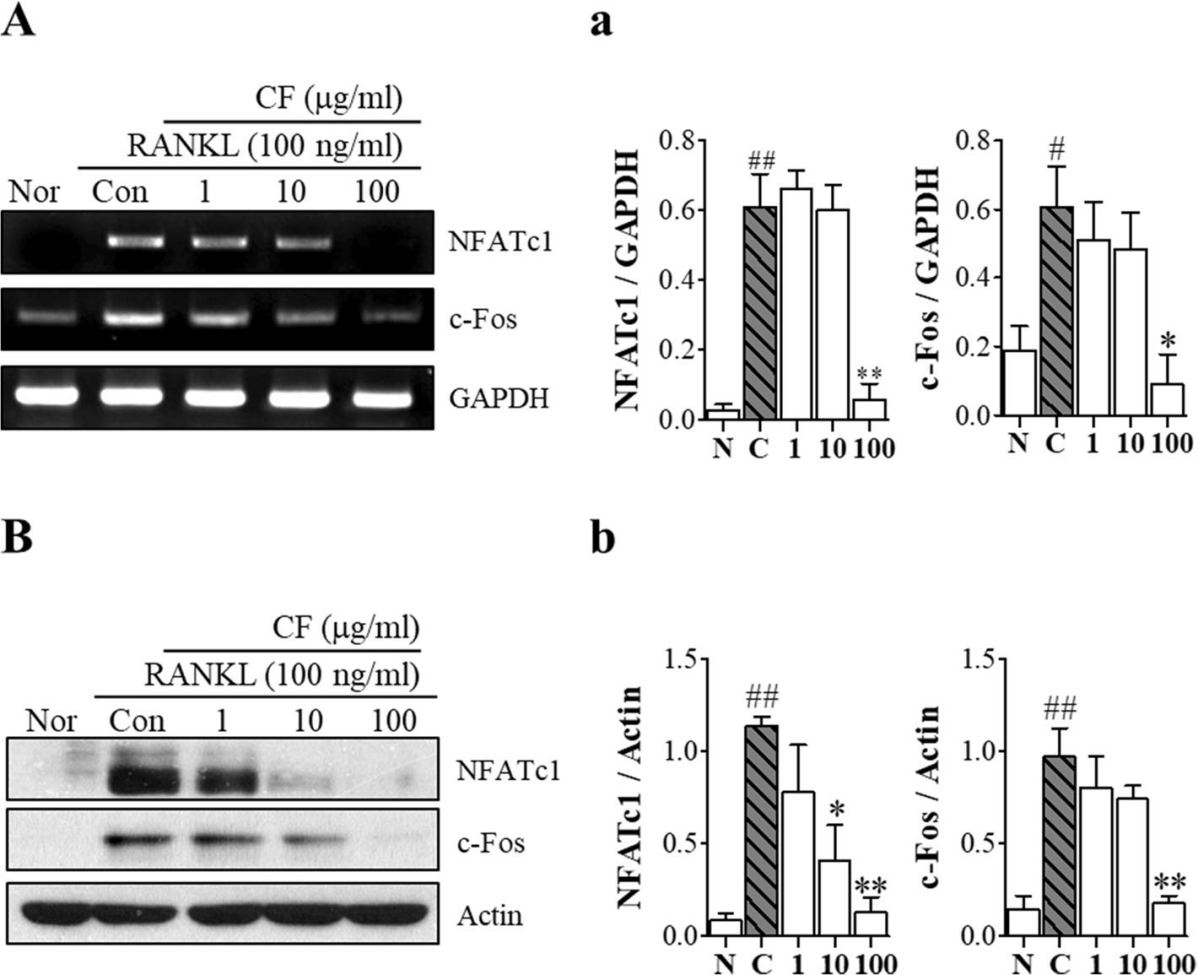
The effects of CF on NFATc1 and c-Fos expression. RAW 264.7 cells were treated with RANKL (100 ng/ml) and CF (1, 10, 100 μg/ml) for 4 days. **A** NFATc1 and c-Fos mRNA expression was detected using RT-PCR. **a** mRNA expression was normalized to GAPDH. **B** NFATc1 and c-Fos protein levels were determined by western blot. **b** Protein expression was normalized to GAPDH and actin. Data represent the means ± SEM of three independent experiments. ^##^*P* < 0.01, ^#^*P* < 0.05 compared with normal and ^**^P < 0.01, ^*^P < 0.05 compared with control. NFATc1, nuclear factor of activated T-cells; GAPDH, glyceraldehyde 3-phosphate dehydrogenase; Nor, nontreated; Con, RANKL-treated control

Next, we confirmed the expression of NFATc1 and c-Fos which are master regulators of osteoclast differentiation. CF inhibited the mRNA expression of NFATc1 (*P* < 0.01) and c-Fos (*P* < 0.05) (Fig. 4A and a) and the proteins expression levels of the corresponding NFATc-1 (*P* < 0.01) and c-Fos (*P* < 0.01) were decreased by CF (Fig. 4B and b).

### CF decreases RANKL-induced expression of osteoclast-related genes and protein

**Fig. 5:**
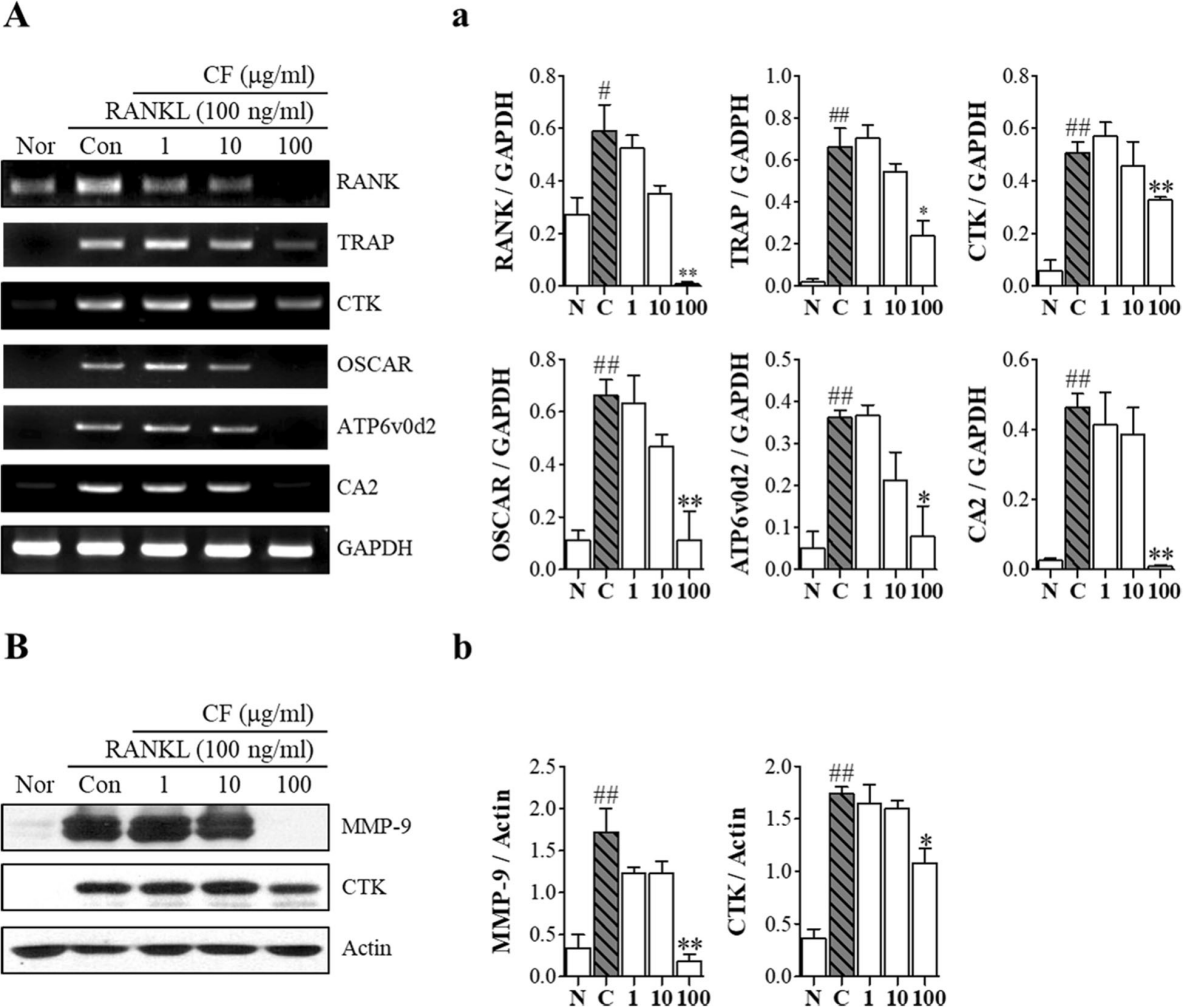
The effect of CF on the expression of osteoclastogenesis-related genes. RAW 264.7 cells were treated with RANKL (100 ng/ml) and CF (1, 10, 100 μg/ml) for 4 days. **A** RANK, TRAP, CTK, OSCAR, ATP6v0d2 and CA2 mRNA expression levels were detected using RT-PCR. **a** The mRNA expression was normalized to GAPDH. **B** CTK and MMP-9 protein expression levels were detected using western blot. **b** Protein expression was normalized to actin. Data represent the means ± SEM of three independent experiments. ^##^*P* < 0.01, ^#^*P* < 0.05 compared with normal and ^**^P < 0.01, ^*^P < 0.05 compared with control; RANK, receptor activator nuclear factor-κB; TRAP, tartrate-resistant acid phosphatase; CTK, cathepsin K; OSCAR, osteoclast-associated receptor; ATP6v0d2, ATPase H+ transporting V0 Subunit D2; CA2, Carbonic anhydrase II; GAPDH, glyceraldehyde 3-phosphate dehydrogenase; MMP-9, Matrix metallopeptidase 9; N, nontreated; C, RANKL-treated control

To further confirm the inhibitory effect of osteoclast differentiation, the effects of CF on the osteoclast-related genes were confirmed by RT-PCR detection of RANK, TRAP, CTK, OSCAR, ATP6v0d2 and CA2 expression (Fig. 5A). CF decreased the expression levels of osteoclast differentiation-related genes in a dose-dependent manner (Fig. 5a). Then, we examined whether CF regulates the expression of bone resorption-related enzymes (Fig. 5B). The protein levels of MMP-9 (*P* < 0.01) and CTK (*P* < 0.05) were also inhibited by CF treatment (Fig. 5b).

### CF inhibits bone loss and serum level of TRAP activity and ALP

**Fig. 6:**
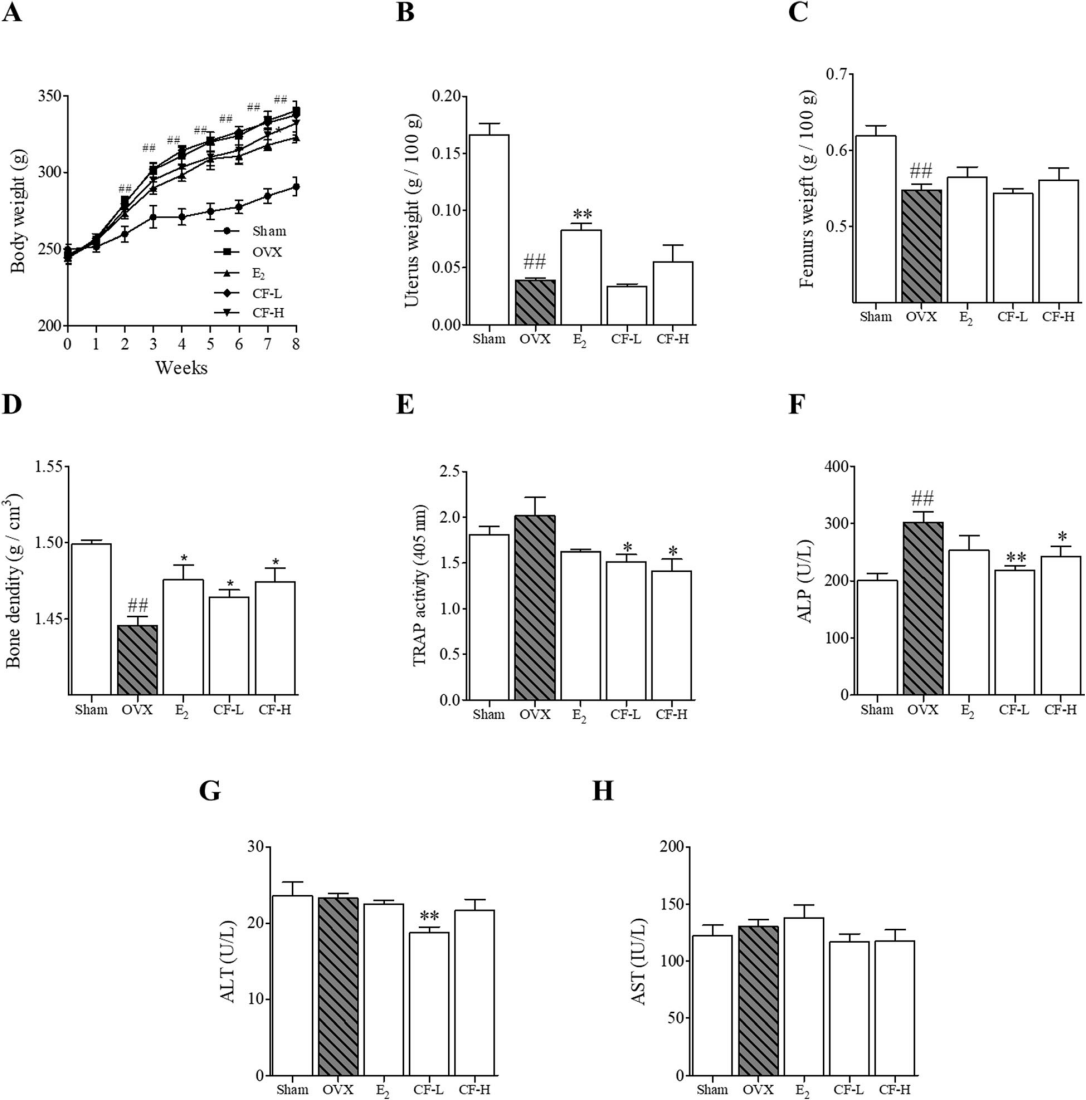
The effect of CF on OVX-induced osteoporosis rats. **A** Weights of rats were measured weekly, and the rats were sacrificed after weighing the **B** uterus and **C** femur. **D** Femur bone density was measured using Archimedes’ principle. CF treatment suppressed the bone metabolism indicator of OVX-induced rats in serum. **E** serum TRAP activity; **F** ALP; **G** ALT; **H** AST. Data represent as mean ± SEM (*n* = 8). ^##^*P* < 0.01 compared with sham and ^**^P < 0.01, ^*^P < 0.05 compared with OVX. TRAP, tartrate-resistant acid phosphatase; ALP, alkaline phosphatase; ALT, alanine transaminase; AST, aspartate aminotransferase; Sham, sham-operated and vehicle-treated group; OVX, bilateral-ovariectomized and vehicle-treated group; E_2,_ bilateral-ovariectomized and 17β-estradiol-treated group; CF-L, bilateral-ovariectomized and low-dose of Chaenomelis fructus-treated group (35 mg/kg); CF-H, high-dose of bilateral-ovariectomized and Chaenomelis fructus-treated group (350 mg/kg)

As shown in Fig. 6a, after OVX, the CF group did not show an effect on body weight, and the E_2_ group showed significantly inhibited weight gain at 8 weeks (*P* < 0.05). In addition, treatment with CF did not show any effect on uterine and femur weight loss due to estrogen deficiency (Fig. 6b and c). Bone loss in the femoral head was observed in the OVX-group. The CF-treated group significantly inhibited this reduction (*P* < 0.05) (Fig. 6d). TRAP activity of serum was increased due to OVX. But the difference was not significant (Fig. 6e). However, the CF-treated group inhibited the expression of TRAP activity (*P* < 0.05). The ALP in serum were significantly increased by OVX, and this increase was confirmed to be reduced through the administration of low and high group of CF (*P* < 0.01, *P* < 0.05) (Fig. 6f). As shown in Fig. 6g and h, Serum ALT and AST levels did not change in SD-rats significantly. Specifically, the low concentration CF dose group significantly inhibited the expression of ALT (*P* < 0.01).

### CF inhibits histological changes and osteoclast differentiation by suppressing expression of NFATc1 in the femur

**Fig. 7:**
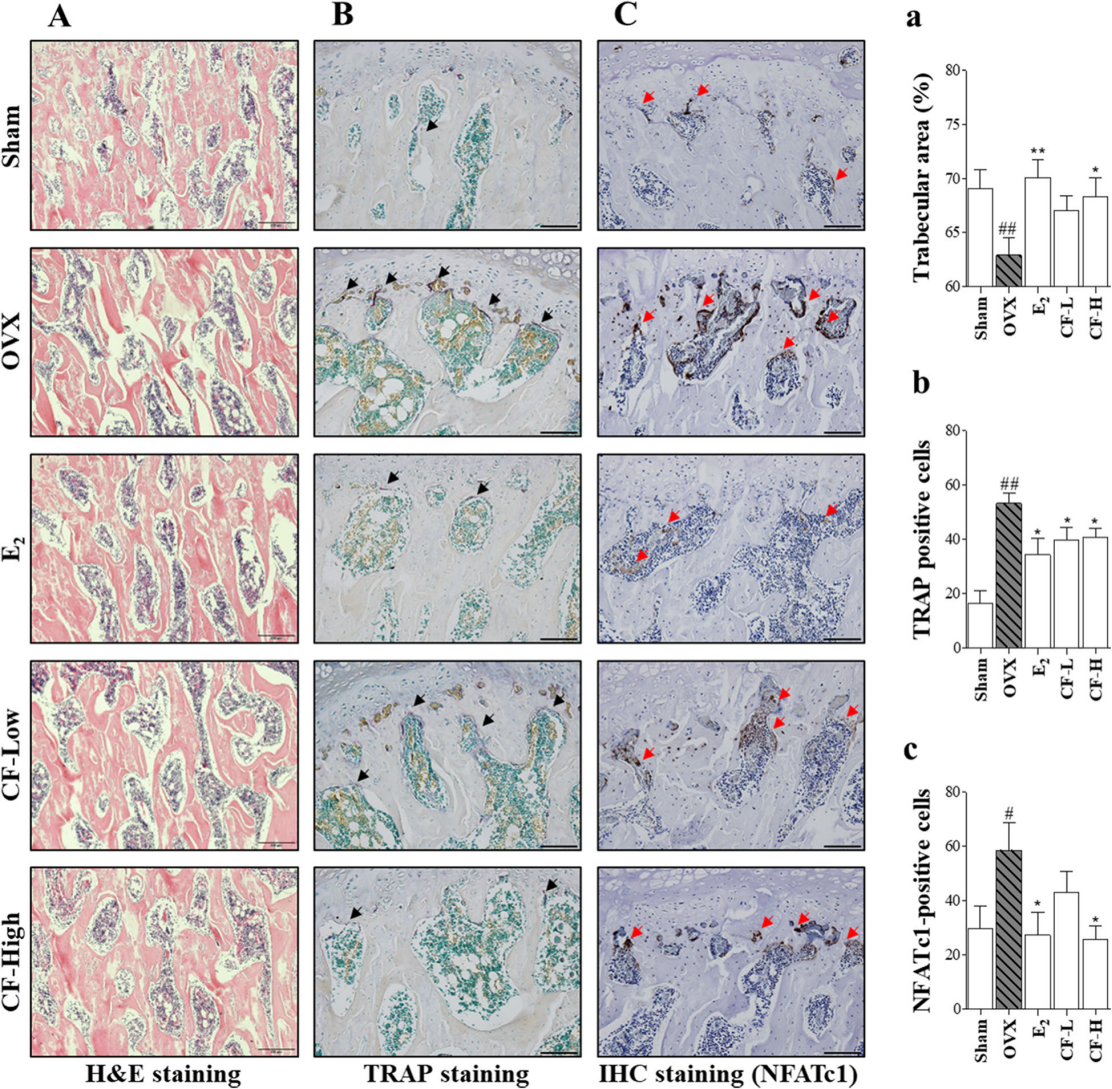
Histological examination of OVX-induced femurs. **a** Representative optical images of H&E staining of OVX-induced femurs from each group. **a** Trabecular area was measured using ImageJ software. **b** TRAP staining of femurs; black arrows indicate areas of TRAP-positive multinuclear cells. **b** Quantification of TRAP-positive cells. **c** Immunohistochemistry staining of NFATc1 in femur; red arrows indicate areas of NFATc1-positive cells. **c** Quantification of NFATc1-positive cells. All images were captured with a light microscope. (200×, Scale bar: 100 μm) Data represent as mean ± SEM (n = 8). ^##^*P* < 0.01, ^#^*P* < 0.05 compared with sham and ^**^P < 0.01, ^*^P < 0.05 compared with OVX. Sham, sham-operated and vehicle-treated group; OVX, bilateral-ovariectomized and vehicle-treated group; E_2,_ bilateral-ovariectomized and 17β-estradiol-treated group; CF-L, bilateral-ovariectomized and low-dose of Chaenomelis fructus-treated group (35 mg/kg); CF-H, high-dose of bilateral-ovariectomized and Chaenomelis fructus-treated group (350 mg/kg); NFATc1, nuclear factor of activated T-cells

The femoral tissues of the OVX-induced osteoporosis rats were investigated for histological changes by H&E staining (Fig. 7A). The decrease of bone density in the OVX rats was confirmed by the reduction of the trabecular area (*P* < 0.01). The CF-H and E_2_ groups showed significantly inhibited trabecular areas (*P* < 0.05, *P* < 0.01) (Fig. 7a). Next, femoral tissue was subjected to TRAP staining to identify osteoclasts that play an important role in reducing the trabecular area (Fig. 7B). TRAP-positive cells were increased in the femoral tissue after OVX (*P* < 0.01). However, the CF-treated group inhibited the number of TRAP-positive cells in the femoral tissue (*P* < 0.05) (Fig. 7b). In addition, we verified the expression of NFATc1, which plays an important role in osteoclast differentiation in femoral tissue (Fig. 7C). Consistent with the TRAP staining results, NFATc1-positive cells were increased in the femoral tissue after OVX (*P* < 0.05), and the CF-treated group inhibited the number of NFATc1-positive cells in the femoral tissue (*P* < 0.05) (Fig. 7c).

## Discussion

In this study, we demonstrated that CF suppressed osteoclast differentiation and resorptive activity through inhibition of NFATc1 and c-Fos, master regulators of osteoclast differentiation. Consistent with in vitro results, CF also inhibited bone loss in OVX-induced rats, which is representative of a postmenopausal osteoporosis model. TRAP secreted by differentiated osteoclasts is regarded as a phenotype of osteoclasts [23] and is responsible for osteoclast migration and bone resorption [18]. In addition, in the pit formation assay, resorption lacunae and area represent the function of osteoclasts. Our study showed that CF treatment inhibited the TRAP-positive cells and their activity; CF treatment also decreased the resorbed pits and area. These results indicate that CF not only inhibited osteoclast differentiation but also suppressed its function.

When RANKL binds to RANK, it immediately activates TRAF6 adaptor protein, resulting in a series of downstream signal cascades that regulate osteoclast differentiation and activation [24]. NF-κB is well known as an important osteoclast differentiation transcription factor. Stimulation due to TRAF6 phosphorylates NF-κB and causes translocation from the cytoplasm to the nucleus [25]. These results upregulate the expression of various transcription genes such as NFATc1, which are important for osteoclast differentiation. In our study, CF not only inhibited the expression of TRAF6, but also suppressed translocation of NF-κB from the cytoplasm to the nucleus. The activity of TRAF6-dependent mediated MAPKs has proved its importance in various studies. In spleen cells deficient in TRAF6, p38 and JNK are not activated despite RANKL stimulation [26]. In Huang et al. study, treatment of the p38 inhibitor SB203580 confirmed that the expression of NFATc1 and c-Fos was suppressed in osteoclast progenitor cells [27]. In addition, osteoclast progenitor cells extracted from jnk1-lacking mice had reduced osteoclast differentiation ability [28]. phosphorylation of ERK is implicated in the differentiation, formation, proliferation and apoptosis of osteoclasts [29]. In this study, CF strongly inhibited the phosphorylation of JNK and p38. These results indicate that the efficacy of CF to inhibit the expression of NFATc1 and c-Fos is because it inhibits the expression of TRAF and NF-kB and the phosphorylation of JNK and p38 which reacts immediately after RANKL stimulation.

Other studies have demonstrated that NFATc1 deficiency causes osteopetrosis [30], indicating that NFATc1 is a crucial factor for osteoclast differentiation. In addition, overexpression of NFATc1 makes osteoclast precursor cells differentiable, even in the absence of RANKL [30]. NFATc1 regulates the expression of various osteoclast-related genes, such as TRAP, CTK, MMP-9, OSCAR and ATP6v0d2 [30–32]. As is shown in our study, CF treatment inhibited the expression of NFATc1 at the mRNA and protein levels. In previous studies, c-Fos mutant mice were used to demonstrate that osteoclasts failed to absorb bone [8] and c-Fos induces the expression of CA2, which plays an important role in acidifying bone surfaces when osteoclasts absorb bone [33]. In addition, various studies have reported that c-Fos expression is involved in NFATc1 expression [34]. In our study, CF treatment suppressed the expression of c-Fos at the mRNA and protein levels. Moreover, we have identified indicators related to osteoclast differentiation, fusion and bone resorption. OSCAR is a component of the immunoglobulin-like surface receptor family, and OSCAR signaling improves the induction of RANKL-mediated NFATc1 through calcium activation [35]. In this experiment, CF inhibited OSCAR expression. This result indicates that CF suppresses the extra mechanism of NFATc1 induction. ATP6v0d2 expression is essential when fused from osteoclast precursor cells to multinuclear osteoclasts. In previous studies, deficiency of ATP6v0d2 in mice has been shown to impair osteoclastogenesis [36]. In our study, CF significantly inhibited gene expression of ATP6v0d2. These results suggest that CF inhibits the fusion of osteoclast precursor cells. The osteoclasts secrete enzymes on the bone surface to promote bone resorption. CA2 affects osteoclast differentiation and acts to help other enzymes function by acidifying the bone surface during bone resorption. The acidified bone surface is degraded by CTK and MMP-9 expression. In particular, CTK is specifically expressed in mature osteoclasts, similar to TRAP. For this reason, CTK expression can be regarded as the number of osteoclasts [37]. In our study, CF inhibited CA2, MMP-9 and CTK expression. These results indicate that CF inhibits the expression of factors related to bone resorption through inhibiting expression of NFATc1 and c-Fos (Additional file 1: Figure S1).

The osteoporotic rat model of OVX was used to confirm the anti-osteoporosis model of CF. Atrophy of the uterus determines the success of OVX [38]. This is because estrogen has a direct effect on the weight of the uterus; also, OVX causes weight gain, and these changes are reversed by E_2_ treatment [20]. In this experiment, OVX showed a decrease in uterine weight and an increase in body weight, and CF did not affect the changes in body weight and uterine weight. The results indicate that CF did not show estrogen-like effects. Estrogen deficiency due also affects various bone metabolic factors. OVX induced abnormal overexpression of osteoclast, which increased the expression of TRAP activity in serum. In this study, no significant difference was found between the Sham and OVX groups. This result is presumably due to the long duration of osteoporosis induction after OVX. Miyazaki et al. reported that more differences in TRAP activity expression were observed in experiments less than eight weeks [39]. Nevertheless, the administration of CF significantly inhibited TRAP activity in serum, which is the same as the results of cell experiments and histochemical tests. ALP is commonly used as a marker for measuring osteoblast activity. The reason why OVX increases expression of ALP is to compensate for the excessive activity of osteoclasts [40]. In our study, CF significantly inhibited the expression of ALP in the serum. These results indicate that CF has a positive effect on OVX-induced bone metabolism markers. ALT and AST represent hepatocellular damage and are very sensitive to hepatotoxicity. This is considered a preclinical and clinical biomarker [41]. In this experiment, ALT and AST showed no significant change. Rather, low concentrations of CF have been shown to inhibit the expression of ALT. These results indicate that concerns about toxicity due to CF dosing in this animal experiment have been eliminated.

Postmenopausal osteoporosis reduces bone quality by reducing bone density and bone area [38]. This is a result of abnormal osteoclast activity, and inhibition of osteoclast differentiation is the most important strategy for the treatment of osteoporosis. In this study, bone mineral density and trabecular area decreased after OVX. The high-dose CF group showed a significant inhibitory effect on the reduction of bone mineral density and trabecular area. Moreover, CF was also effective in reducing the number of osteoclasts and inhibiting NFATc1 expression. These results suggest that CF may be an alternative treatment for postmenopausal osteoporosis through inhibition of osteoclast differentiation and inhibition of bone loss.

Representative ingredient of CF are quinic acid, malic acid, shikimic acid, protocatechuic acid and chlorogenic acid [42]. These ingredients have demonstrated various pharmacological effects on osteoclasts and osteoporosis. The study by Silva et al. demonstrated that low concentrations of quinic acid strongly inhibited osteoclast activity [43]. Shikimic Acid suppress osteoclast differentiation by inhibiting RANK/TRAF6 and Suppressing NF-κB and MAPK signaling pathways [44]. These results are very similar to the osteoclast suppression mechanism of CF. Wu’s research demonstrated that protocatechuic acid inhibits osteoclast differentiation and induces apoptosis in mature osteoclasts [45]. Kwak et al. reported that chlorogenic acid inhibits osteoclast differentiation by decreasing NFATc1 expression [46]. These results can be indicated that CF’s osteoclast differentiation inhibitory effect and osteoporosis protective effect are due to the pharmacological action of the main ingredients.

This study has the following limitations: i) Normal osteoclasts require RANKL and M-CSF when they differentiate, but RAW 264.7 cells do not require M-CSF. This cannot be explained by the complete analysis of physiological osteoclast differentiation [47]. Therefore, it is necessary to evaluate the osteoclast inhibition ability of CF through bone marrow macrophages cells extracted from mice or rats under the RANKL and M-CSF treatment in the future. ii) In this study, CF had a positive effect on bone density using Archimedes principle. In addition, in tissue staining, CF reduced the trabecular area loss and the number of TRAP-positive cells. However, this is relatively less reliable than bone architecture analysis using micro-CT [48]. In the future, changes in bone density through the administration of CF with micro-CT analysis should be studied.

## Conclusion

In summary, we have demonstrated that CF has the potential to suppress osteoclast differentiation through regulation of the TRAF6/NF-κB/MAPK/NFATc1 signaling pathway. In addition, CF inhibited bone loss in osteoporosis rats caused by estrogen deficiency-induced. The effect of CF may be a potential alternative to the treatment of osteoporosis without inducing estrogen-like proliferative effects and hepatotoxicity.

## Supplementary information

**Additional file 1.** Mechanisms for the anti-osteoclastogenesis effect of CF in RANKL-induced RAW 264.7 cells. CF suppresses osteoclast differentiation and its activity via inhibiting expression of NFATc1 and osteoclastogenesis-related markers. CF inhibited the expression of RANKL-stimulated TRAF6, MAPK and NF-kB. As a result, CF inhibited the expression of NFATc1 and c-Fos, which are key markers for osteoclast differentiation. Finally, CF inhibits the expression of various osteoclast-related genes such as CA2, MMP-9, CTK, TRAP, ATP6v0d2 and OSCAR.

## Data Availability

All data generated or analysed during this study are included in this published article. [and its supplementary information files].

## Abbreviations

ALP: Alkaline phosphatase
ALT: Alanine transaminase
AST: Aspartate aminotransferase
CF: Chaenomelis fructus
CTK: Cathepsin K
ECL: Enhanced chemiluminescence
ERK: Extracellular-signal-regulated kinase
GAPDH: Glyceraldehyde 3-phosphate dehydrogenase
H&E: Hematoxylin–eosin
HPLC: High performance liquid chromatography
IHC: Immunohistochemistry
JNK: c-Jun N-terminal kinases
MAPKs: Mitogen-activated protein kinase
MMP-9: Matrix metallopeptidase-9
NF- κB: Nuclear factor-kappa B
NFATc1: Nuclear factor of activated T-cells, cytoplasmic 1
OSCAR: Osteoclast-associated immunoglobulin-like receptor
ATP6v0D2: ATPase, H+ transporting, lysosomal 38 kDa, V0 subunit d2
CA2: Carbonic anhydrase 2
OVX: Ovariectomized
RANK: Receptor activator of the nuclear factor kappa B
RANKL: Receptor activator of the nuclear factor kappa B ligand
TRAF6: Tumor necrosis factor receptor-associated factor 6
TRAP: Tartrate-resistant acid phosphatase

## References

[1] Sozen T, Ozisik L, Basaran NC (2017). An overview and management of osteoporosis. Eur J Rheumatol.

[2] Heaney RP (1998). Pathophysiology of osteoporosis. Endocrinol Metab Clin N Am.

[3] Walsh MC, Kim N, Kadono Y, Rho J, Lee SY, Lorenzo J, Choi Y (2006). Osteoimmunology: interplay between the immune system and bone metabolism. Annu Rev Immunol.

[4] de Villiers TJ (2015). The role of menopausal hormone therapy in the management of osteoporosis. Climacteric.

[5] Bar-Shavit Z (2007). The osteoclast: a multinucleated, hematopoietic-origin, bone-resorbing osteoimmune cell. J Cell Biochem.

[6] Boyce BF, Xing L (2008). Functions of RANKL/RANK/OPG in bone modeling and remodeling. Arch Biochem Biophys.

[7] Boyle WJ, Simonet WS, Lacey DL (2003). Osteoclast differentiation and activation. Nature.

[8] Grigoriadis AE, Wang ZQ, Cecchini MG, Hofstetter W, Felix R, Fleisch HA, Wagner EF (1994). C-Fos: a key regulator of osteoclast-macrophage lineage determination and bone remodeling. Science.

[9] Zhao Q, Wang X, Liu Y, He A, Jia R (2010). NFATc1: functions in osteoclasts. Int J Biochem Cell Biol.

[10] David JP, Rincon M, Neff L, Horne WC, Baron R (2001). Carbonic anhydrase II is an AP-1 target gene in osteoclasts. J Cell Physiol.

[11] Fujisaki K, Tanabe N, Suzuki N, Kawato T, Takeichi O, Tsuzukibashi O, Makimura M, Ito K, Maeno M (2007). Receptor activator of NF-kappaB ligand induces the expression of carbonic anhydrase II, cathepsin K, and matrix metalloproteinase-9 in osteoclast precursor RAW264.7 cells. Life Sci.

[12] Kennel KA, Drake MT (2009). Adverse effects of bisphosphonates: implications for osteoporosis management. Mayo Clin Proc.

[13] Giannakeas V, Cadarette SM, Ban JK, Lipscombe L, Narod SA, Kotsopoulos J. Denosumab and breast cancer risk in postmenopausal women: a population-based cohort study. Br J Cancer. 2018. 10.1038/s41416-018-0225-4 PMC6265331 30420611

[14] Bodenner D, Redman C, Riggs A (2007). Teriparatide in the management of osteoporosis. Clin Interv Aging.

[15] Persson I, Weiderpass E, Bergkvist L, Bergstrom R, Schairer C (1999). Risks of breast and endometrial cancer after estrogen and estrogen-progestin replacement. CCC.

[16] Herbology Editorial Committee of Korean Medicine: Herbology. Younglimsa, Seoul, 2004 (In Korean).

[17] Chen Q, Wei W (2003). Effects and mechanisms of glucosides of chaenomeles speciosa on collagen-induced arthritis in rats. Int Immunopharmacol.

[18] Takayanagi H (2007). Osteoimmunology: shared mechanisms and crosstalk between the immune and bone systems. Nat Rev Immunol.

[19] Tanabe N, Maeno M, Suzuki N, Fujisaki K, Tanaka H, Ogiso B, Ito K (2005). IL-1 alpha stimulates the formation of osteoclast-like cells by increasing M-CSF and PGE2 production and decreasing OPG production by osteoblasts. Life Sci.

[20] Kalu DN (1991). The ovariectomized rat model of postmenopausal bone loss. Bone Mineral.

[21] Miao J, Zhao C, Li X, Chen X, Mao X, Huang H, Wang T, Gao W (2016). Chemical composition and bioactivities of two common Chaenomeles fruits in China: Chaenomeles speciosa and Chaenomeles sinensis. J Food Sci.

[22] Collin-Osdoby P, Yu X, Zheng H, Osdoby P (2003). RANKL-mediated osteoclast formation from murine RAW 264.7 cells. Methods Mol Med.

[23] Hsu H, Lacey DL, Dunstan CR, Solovyev I, Colombero A, Timms E, Tan HL, Elliott G, Kelley MJ, Sarosi I (1999). Tumor necrosis factor receptor family member RANK mediates osteoclast differentiation and activation induced by osteoprotegerin ligand. Proc Natl Acad Sci U S A.

[24] Armstrong AP, Tometsko ME, Glaccum M, Sutherland CL, Cosman D, Dougall WC (2002). A RANK/TRAF6-dependent signal transduction pathway is essential for osteoclast cytoskeletal organization and resorptive function. J Biol Chem.

[25] Soysa NS, Alles N (2009). NF-kappaB functions in osteoclasts. Biochem Biophys Res Commun.

[26] Kobayashi N, Kadono Y, Naito A, Matsumoto K, Yamamoto T, Tanaka S, Inoue J (2001). Segregation of TRAF6-mediated signaling pathways clarifies its role in osteoclastogenesis. EMBO J.

[27] Huang H, Chang EJ, Ryu J, Lee ZH, Lee Y, Kim HH (2006). Induction of c-Fos and NFATc1 during RANKL-stimulated osteoclast differentiation is mediated by the p38 signaling pathway. Biochem Biophys Res Commun.

[28] David JP, Sabapathy K, Hoffmann O, Idarraga MH, Wagner EF (2002). JNK1 modulates osteoclastogenesis through both c-Jun phosphorylation-dependent and -independent mechanisms. J Cell Sci.

[29] Lee Kyunghee, Seo Incheol, Choi Mun, Jeong Daewon (2018). Roles of Mitogen-Activated Protein Kinases in Osteoclast Biology. International Journal of Molecular Sciences.

[30] Kim JY, Cheon YH, Yoon KH, Lee MS, Oh J (2014). Parthenolide inhibits osteoclast differentiation and bone resorbing activity by down-regulation of NFATc1 induction and c-Fos stability, during RANKL-mediated osteoclastogenesis. BMB Rep.

[31] Kang TH, Baek HY, Kim YC (2005). Protective effect of jakyak-gamcho-tang extract and its constituents against t-BHP-induced oxidative damage in HT22 cells. Am J Chin Med.

[32] Kim K, Lee SH, Ha Kim J, Choi Y, Kim N (2008). NFATc1 induces osteoclast fusion via up-regulation of Atp6v0d2 and the dendritic cell-specific transmembrane protein (DC-STAMP). Mol Endocrinol.

[33] Biskobing DM, Fan D, Fan X, Rubin J (1997). Induction of carbonic anhydrase II expression in osteoclast progenitors requires physical contact with stromal cells. Endocrinology.

[34] Kim SB, Ahn B, Kim M, Ji HJ, Shin SK, Hong IP, Kim CY, Hwang BY, Lee MK (2014). Effect of Cordyceps militaris extract and active constituents on metabolic parameters of obesity induced by high-fat diet in C58BL/6J mice. J Ethnopharmacol.

[35] Kim JH, Kim K, Jin HM, Youn BU, Song I, Choi HS, Kim N (2008). Upstream stimulatory factors regulate OSCAR gene expression in RANKL-mediated osteoclast differentiation. J Mol Biol.

[36] Lee SH, Rho J, Jeong D, Sul JY, Kim T, Kim N, Kang JS, Miyamoto T, Suda T, Lee SK (2006). V-ATPase V0 subunit d2-deficient mice exhibit impaired osteoclast fusion and increased bone formation. Nat Med.

[37] Henriksen K, Tanko LB, Qvist P, Delmas PD, Christiansen C, Karsdal MA (2007). Assessment of osteoclast number and function: application in the development of new and improved treatment modalities for bone diseases. Osteoporos Int.

[38] Barlet JP, Coxam V, Davicco MJ, Gaumet N (1994). Animal models of post-menopausal osteoporosis. Reprod Nutr Dev.

[39] Miyazaki T, Matsunaga T, Miyazaki S, Hokari S, Komoda T (2004). Changes in receptor activator of nuclear factor-kappaB, and its ligand, osteoprotegerin, bone-type alkaline phosphatase, and tartrate-resistant acid phosphatase in ovariectomized rats. J Cell Biochem.

[40] Kuo TR, Chen CH (2017). Bone biomarker for the clinical assessment of osteoporosis: recent developments and future perspectives. Biomark Res.

[41] Ozer J, Ratner M, Shaw M, Bailey W, Schomaker S (2008). The current state of serum biomarkers of hepatotoxicity. Toxicology.

[42] Zhu L, Fang L, Li Z, Xie X, Zhang L (2019). A HPLC fingerprint study on Chaenomelis Fructus. BMC Chem.

[43] Silva MRMA, Pacheco CMF, Madeira MFM, Saraiva AM, Freitas EA, Valverde TM, Gomes JHS, Pádua RM, Kitten GT, Alves SYF (2019). Effect of the extract and constituents from Hancornia speciosa fruits in osteoclasts. Planta Med.

[44] Chen X, Li X, Zhai X, Zhi X, Cao L, Qin L, Su J (2018). Shikimic acid inhibits Osteoclastogenesis in vivo and in vitro by blocking RANK/TRAF6 association and suppressing NF-kappaB and MAPK signaling pathways. Cell Physiol Biochem.

[45] Wu YX, Wu TY, Xu BB, Xu XY, Chen HG, Li XY, Wang G (2016). Protocatechuic acid inhibits osteoclast differentiation and stimulates apoptosis in mature osteoclasts. Biomed Pharmacother.

[46] Kwak SC, Lee C, Kim JY, Oh HM, So HS, Lee MS, Rho MC, Oh J (2013). Chlorogenic acid inhibits osteoclast differentiation and bone resorption by down-regulation of receptor activator of nuclear factor kappa-B ligand-induced nuclear factor of activated T cells c1 expression. Biol Pharm Bull.

[47] Ng AY, Tu C, Shen S, Xu D, Oursler MJ, Qu J, Yang S (2018). Comparative characterization of osteoclasts derived from murine bone marrow macrophages and RAW 264.7 cells using quantitative proteomics. JBMR Plus.

[48] Adams GJ, Cook RB, Hutchinson JR, Zioupos P. Bone Apparent and Material Densities Examined by Cone Beam Computed Tomography and the Archimedes Technique: Comparison of the Two Methods and Their Results. Front Mech Eng. 2018:3(23).

