# Supplementary figures and images for: Chaenomelis fructus inhibits osteoclast differentiation by suppressing NFATc1 expression and prevents ovariectomy-induced osteoporosis
Source: BMC Complement Med Ther. 2020 Feb 5;20:35. doi: 10.1186/s12906-020-2841-9 (PMC7076887; doi:10.1186/s12906-020-2841-9)

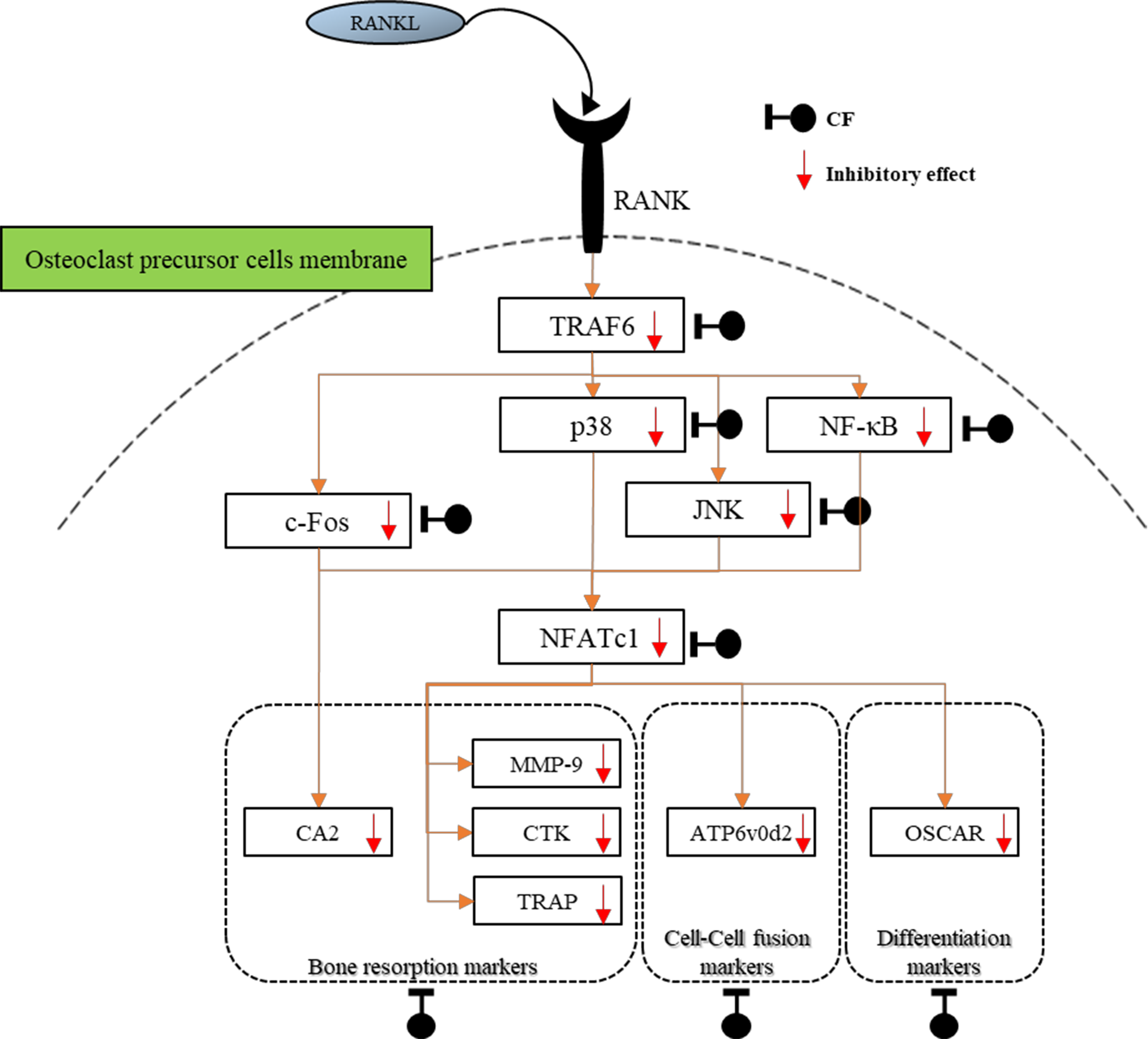

Supplement: Supplementary file 1 — Additional file 1. Mechanisms for the anti-osteoclastogenesis effect of CF in RANKL-induced RAW 264.7 cells. CF suppresses osteoclast differentiation and its activity via inhibiting expression of NFATc1 and osteoclastogenesis-related markers. CF inhibited the expression of RANKL-stimulated TRAF6, MAPK and NF-kB. As a result, CF inhibited the expression of NFATc1 and c-Fos, which are key markers for osteoclast differentiation. Finally, CF inhibits the expression of various osteoclast-related genes such as CA2, MMP-9, CTK, TRAP, ATP6v0d2 and OSCAR. [file 12906_2020_2841_MOESM1_ESM.tif]
